# Tracking the migration of the Indian continent using the carbonate clumped isotope technique on Phanerozoic soil carbonates

**DOI:** 10.1038/srep22187

**Published:** 2016-03-02

**Authors:** Prosenjit Ghosh, Mikhail V. Vasiliev, Parthasarathi Ghosh, Soumen Sarkar, Sampa Ghosh, Keita Yamada, Yuichiro Ueno, Naohiro Yoshida, Christopher J. Poulsen

**Affiliations:** 1 Centre for Earth Sciences, Indian Institute of Sciences, Bangalore, 560012, India; 2 Divecha Centre for Climate Change, Indian Institute of Science, Bangalore, 560012; 3 Geological Studies Unit, Indian Statistical Institute, 203, B.T. Road, Kolkata 700 108, India; 4 Interdisciplinary Graduate School of Science and Engineering, Tokyo Institute of Technology, Yokohama, 226-8502, Japan; 5 Department of Earth and Planetary Sciences, Tokyo Institute of Technology, Meguro, Tokyo 152-8551, Japan; 6 Earth-Life Science Institute, Tokyo Institute of Technology, Meguro, Tokyo 152-8551, Japan; 7 Dept. of Earth and Environmental Sciences, University of Michigan, Ann Arbor, MI 48109-1005.

## Abstract

Approximately 140 million years ago, the Indian plate separated from Gondwana and migrated by almost 90° latitude to its current location, forming the Himalayan-Tibetan system. Large discrepancies exist in the rate of migration of Indian plate during Phanerozoic. Here we describe a new approach to paleo-latitudinal reconstruction based on simultaneous determination of carbonate formation temperature and δ^18^O of soil carbonates, constrained by the abundances of ^13^C-^18^O bonds in palaeosol carbonates. Assuming that the palaeosol carbonates have a strong relationship with the composition of the meteoric water, δ^18^O carbonate of palaeosol can constrain paleo-latitudinal position. Weighted mean annual rainfall δ^18^O water values measured at several stations across the southern latitudes are used to derive a polynomial equation: δ^18^Ow = −0.006 × (LAT)^2^ − 0.294 × (LAT) − 5.29 which is used for latitudinal reconstruction. We use this approach to show the northward migration of the Indian plate from 46.8 ± 5.8°S during the Permian (269 M.y.) to 30 ± 11°S during the Triassic (248 M.y.), 14.7 ± 8.7°S during the early Cretaceous (135 M.y.), and 28 ± 8.8°S during the late Cretaceous (68 M.y.). Soil carbonate δ^18^O provides an alternative method for tracing the latitudinal position of Indian plate in the past and the estimates are consistent with the paleo-magnetic records which document the position of Indian plate prior to 135 ± 3 M.y.

Combined evidence from geomorphology, tomographic images, geochronology, paleontology and lithological indicators provide evidence for the palaeo-latitudinal position of the Indian plate as it was ripped from the Gondwana supercontinent and drifted northward^1^,^2^. However, the strongest evidence comes from the reconstruction of linear magnetic anomalies recovered from sub-aerial flood basalts deposited on continental-rift and sedimentary packets, which preserve information about the paleo-magnetic pole together with the occurrence of climate-sensitive litho-units like coals, evaporates, bauxites and tillites in the sedimentary successions^1^,^2^,^3^,^4^. These paleo-magnetic records disagree in the paleo-position of India during the late Paleozoic and Mesozoic time by up to 30˚ latitude. Reconstructions using this method involve detecting the paleo-magnetic pole from the orientation of the magnetic minerals present in the lava flows^1^. Alternative methods to trace paleo-latitudinal position using stable isotope techniques^5^ suffer from several limitations including diagenetic imprinting, poor understanding of soil temperatures and the influence of evaporation. To refine this technique, we used clumped isotope thermometry to estimate the temperature and isotopic composition of soil water, which is derived primarily measuring the δ^18^O of carbonates^6^. Studies have shown that soil carbonates formed at different latitudes vary in their meteoric water composition^7^. Ancient soils (paleosols) preserve a record of meteoric water isotopic compositions and therefore can be used to trace the latitudinal position during sedimentation. However, the availability of soil carbonates is an important constraint on this method. In general, soil carbonates are found only in arid to sub-humid climatic conditions^1^,^2^ in relatively dry soils where C_3_ type grasses or mixed grasses and shrubs are the dominant vegetation.

The Satpura Basin, located in Central India, has an especially well-preserved record of soil carbonates. Sedimentary sequences comprised of packets of soil carbonate were deposited along the palaeo channel of the present day Narmada River while the Indian plate drifted from its position in the Southern Hemisphere^8^,^9^. These carbonates capture the isotopic signature of ancient soil water, which resemble the isotopic composition of meteoric water. Since soil waters experience evaporation that leads to enrichment in ^18^O, our results place upper limits on meteoric water compositions^5^ unless corrected for the isotopic effect due to evaporation. Lithostratigraphic studies of the Gondwana succession in the Satpura Basin indicate that strata bound calcic vertisols are interspersed in a sequence of mudstones, coarse sandstones with cement carbonaceous shale, coals and bio-fossils^8^. These sedimentary units are found in discrete, discontinuous patches within the 5 km-thick basinal package^10^. Post depositional modification of the sedimentary layers was minimal due to the small overburden of sedimentary piles and was restricted to only early diagenetic transformation^9^,^11^. The sedimentary succession of Satpura was deposited in a mega half-graben confined by faults. Sediment accumulation took place during fault-controlled subsidence regimes with intervening period of tectonic quiescence supported development of soils. The subsidence rate varied across the basin resulting in an asymmetric basin fill with the thickness increasing towards the north^10^. Sedimentation in the basin commenced with deposition of the Permo-Carboniferous (290 M.y. ago) Talchir bed followed by deposition of other sedimentary formations namely the Motur, Pachmarhi, Denwa, Bagra and Lameta with ages varying from 269, 248, 243, 135 and 68 M.y. ago respectively^8^. The focus of the present study is to use the combined temperature and δ^18^O of the carbonates deposited in this succession of fluvial sediments as a geochemical indicator of the palaeo-latitudinal position of the Indian plate.

Age assignments to the soil carbonates are based on biostratigraphic methods as well as relative age dating based on of the position of strata in the lithological section. The presence of vertebrate fossils in the litho-unit and the well-constrained geochronological age of the overlying Deccan basalt, allowed precise assignment of litho and biostratigraphic age to the litho-unit^8^,^9^,^10^,^11^,^12^,^13^. The overlying sequence of Deccan basalt restricted water-rock interaction creating an environment for excellent preservation of these soil archives^8^.

## Clumped isotope analyses

**Figure 1:**
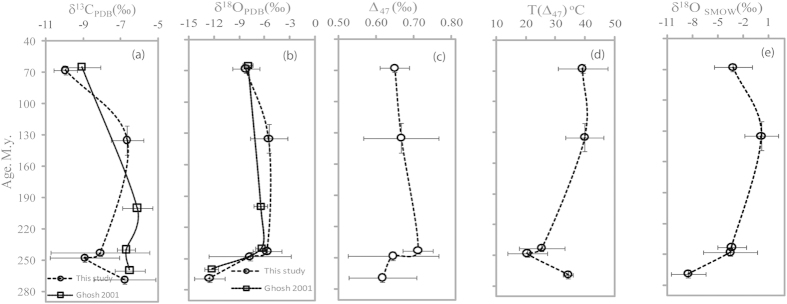
Plots of carbon, oxygen isotope ratios, Δ_47_, temperatures of carbonate precipitation and estimated water isotopic composition with age (time of deposition) are shown. (**a**) Average carbon isotope ratios from replicate samples, (**b**) Average oxygen isotope ratios from replicate samples, and (**c**) Average Δ_47_ measured from replicate samples of soil carbonates obtained from the different strata of varying ages with error bar marking 1σ around mean values. Data from earlier work^8^,^15^,^16^,^17^,^18^ are also shown for comparison (**d**) Average temperature estimates for soil carbonate precipitation estimated from the measured Δ_47_ values by using the relationship given by Ghosh et al.^14^ and later revised by Dennis *et al*., (2012) (**e**) Estimated δ^18^O of soil water in an equilibrium condition plotted with age. Temperature and δ^18^O of carbonate measured in this study were used as input parameters in the oxygen isotope thermometry^18^ equation to obtain the values.

In order to understand the temperature and fluid composition during precipitation of the soil carbonates, we analyzed clumped isotopic ratios in bulk carbonate samples. XRD analysis reveals that calcite and clays are the prominent minerals present in the samples^5^. The abundance of Δ_47,_ δ^13^C and δ^18^O of carbonates from the Motur, Denwa, Pachmarhi, Bagra and Lameta formations are given in Table 1 and Fig. 1. The Δ_47_ constrains carbonate growth temperature independent of the δ^18^O of waters from which they grew^14^. The Δ_47_ temperatures were then used to constrain the δ^18^O of water^14^ (i.e., because both the growth temperature and the δ^18^O of carbonate are known). Here we determined soil water δ^18^O values upon using conventional paleo-thermometry equation^15^ where temperature (Fig. 1e) of carbonate precipitation and δ^18^O of carbonate (Fig. 1) were derived from the experimental results. This soil water δ^18^O value ranges between 0.25 ± 2.20 (‰, VSMOW) and −8.1 ± 2.0 (‰, VSMOW). Uncertainty or heterogeneity in the composition of carbonate is due to variability in temperature of carbonate precipitation, water, soil respiration and is denoted by 1σ standard deviations from the mean and are marked in the plots (Fig. 1e) of Δ_47_, calcification temperature, δ^13^C and δ^18^O. The age uncertainties were established based on the bio-stratigraphy of the Motur, Pachmarhi, Denwa, Bagra and Lameta formations^16^,^17^,^18^,^19^. Both surface temperature and meteoric water δ^18^O are grossly linked with latitude. The relationship with δ^18^O of meteoric water and latitude is assumed to remain similar through 250 M.y. Factor like continental configuration is expected to influence the heat transfer process and isotopic distribution across latitude. But the simulation experiment conducted with land configuration resembling Permian 250 M.y. produced a thermal mode of circulation, similar to the modern North Atlantic, supporting our argument of grossly similar or slightly different equator-to-pole temperature gradient than the modern day^20^. These factors were responsible governing the seasonal distribution of isotopic ratios in meteoric water across the latitudes^21^. However, there are evidences which support that the yearly average isotopic ratios across the latitude remain more or less similar and comparable to modern day. Experimental evidence of comparable lighter isotopic ratios in the fresh water was detected in the carbonate precipitates from Permian and Carboniferous environment support our view point^22^,^23^.

We used Δ_47_ based temperature estimates as input parameter together with δ^18^O of specific soil carbonates to derive the δ^18^O of soil water. Considering soil water as derived from the meteoric water an additional evaporative effect was introduced^5^. The modification of soil water composition likely varied through evaporation depending upon the latitudinal position. An enrichment factor of 2‰ and 2.5‰ are adopted for the samples with palaeo-latitudinal positions south of 50°S and north of 50°S respectively, to reflect this variation (see Table 1). The values obtained for the meteoric water (δ^18^Oppt) are fed into the polynomial equation for the reconstruction of the latitudinal positions and uncertainties of predicted values vary with Latitudes (Extended Data Figs 1 and 2); with higher accuracy been observed for prediction of positions at higher latitudes. The latitudinal dependence of temperature and δ^18^O are known from the IAEA database covering several stations across the Southern Hemisphere. Independent estimates on the *p*CO_2_ level during carbonate precipitation are established from the δ^13^C values of pedogenic carbonates^8^. To account for the influence of changes in atmospheric *p*CO_2_ on air temperature, we compare our results with those from global climate model (GCM) predictions of air temperatures and isotopes ratios in precipitation over a range of atmospheric *p*CO_2_ levels^24^ (Extended data Fig. 3). Although the simulated values are meant for Cretaceous climate, the zonal average temperatures are reasonable for others periods (within the Paleozoic and early Mesozoic era) as well where the *p*CO_2_ concentration were within 4 to 12 times of the present day atmospheric values.

### Age assignment to the strata

The age of the Satpura Basin paleosols are known to range from 269 to 68 M.y. ago based on the occurrence of index fossils including vertebrate fauna and diatoms. The ages have recently been redefined based on discovery of several vertebrate fossils^12^,^13^. The relative ages of the sedimentary strata were also obtained based on the stratigraphic position of the bed with respect to the strata with known ages. In both cases, the floral and faunal fossil records provided biostratigraphic age estimates to the Motur, Pachmarhi, Denwa, Bagra and Lameta formations. The age of the Talchir Formation, which hosts gritty sandstone, glacial conglomerate and striations, is placed at Permo-Carboniferous (290 M.y. ago), while the Motur, Panchmarhi, Denwa and Lameta Formations were dated Early Middle Permian (269 ± 4 M.y. ago), Late Lower Middle Triassic (248 ± 4 M.y. ago), Early Middle Triassic (243 ± 3 M.y. ago) and Late Cretaceous (68 ± 4 M.y. ago) respectively^9^,^10^,^11^,^12^,^13^. In the absence of any fossil records from the Bagra Formation, several workers have contended that it might be equivalent to or younger in age than Denwa^12^,^13^,^25^. Since the age of the Denwa Formation is considered to be between the late Lower to the Middle Triassic^18^, the age of the Bagra formation has been taken to be around the late Triassic^16^,^17^,^18^. The fact that the Bagra Formation overlies the Denwa in Central India suggests that it is younger than late Triassic but older or equivalent to the late Jurassic-early Cretaceous (135 ± 14 M.y. ago)^19^. The Lameta Formation in Central India is located at the Lameta Ghat on the Narmada River, 15 km southwest of Jabalpur. The ~35 m -thick fluvial sediments of the Lameta Formation transitionally overlie the fluvial sandstone and mudstone of the Cretaceous^8^, Jabalpur Formation and are conformably overlain by the Tertiary Deccan trap basalts.

### Estimation of average air temperature from calcite clumped isotopes

Several empirical formulations have been proposed for deriving temperature (air and calcite growth temperature) from clumped isotope data subsequent to the initial paper on clumped-isotope thermometry^26^,^27^. We report here calcite precipitation temperatures derived using the relationship proposed by Ghosh *et al*.^14^ and revised recently to accommodate the absolute reference frame^26^. The precipitation temperatures of calcite present in the samples are 34.1 ± 2 °C for 269 M.y. ago, 20.3 ± 7 °C for 248 M.y. ago, 25.2 ± 8 °C for 243 M.y. ago, 39.7 ± 6 °C for 135 M.y. ago and 39.0 ± 8 °C for 68 M.y. ago. Clumped isotope studies on modern soil carbonates across northern latitudes provide a method to relate clumped temperature with mean annual air temperature and warmest average monthly temperatures^28^. Using this relationship, the mean annual air temperatures (MAAT) were derived for the past soil carbonates^28^ are 19 ± 2 °C for 269 M.y. ago, 3 ± 7 °C for 248 M.y. ago, 8 ± 8 °C for 243 M.y. ago, 26 ± 6 °C for 135 M.y. ago and 25 ± 8 °C for 68 M.y ago (see Extended Table 2). Similarly, warmest monthly air temperatures estimated based on the equation^28^ for our analysis of samples are 31 ± 2 °C for 269 M.y., 14.6 ± 7 °C for 248 M.y., 20 ± 8 °C for 243 M.y., 33.7 ± 6 °C for 135 M.y. and 36.2 ± 8 °C for 68 M.y. The majority of the temperature estimates are within several degree of modern average annual air temperature across the latitudes except for a few samples from the early Cretaceous and Permian, where the estimated temperature are slightly higher than modern day temperatures at those latitudes. It is possible that higher *p*CO_2_ condition during these time periods were responsible for the warmer temperatures recorded in our samples compared to the present day value. The samples recording lowest temperature and lightest δ^18^O are expected to be most pristine, while samples with anomalously high temperatures are likely preserving signature of recrystallization and burial diagenesis which happens at elevated temperatures. In order to rule out the possibility of diffusion related process resetting the original composition, we compared clumped isotope temperature with the inferred temperature expected during deep burial (Extended Data Fig. 3).

We further compare our results with zonal average surface temperatures simulated using a GCM under a range of *p*CO_2_ values^24^. The temperature values observed in our study at different latitudes are cooler than the simulated annual zonal temperatures. This discrepancy might indicate a seasonal bias to calcite precipitation. Calcite precipitation can happen during the period of water saturation or well-drained condition during a year. Studies have shown that the cold dry period with well-drained soil condition induces high soil respiration which is registered as enriched δ^13^C values in the soil carbonates^29^. In contrast, warm wet conditions dampen the rate of soil respiration, which is registered as lighter δ^13^C values in the soil carbonates^29^. A close inspection of the δ^13^C, δ^18^O and deduced Mean Annual Air Temperature (MAAT) for the samples suggests seasonal deposition of Lameta and Bagra soil calcite (Extended Data Fig. 4). Higher rate of soil respiration, low Eh condition and low input of atmospheric CO_2_ is inferred for Lameta soil while soil formed during Bagra deposition registered a low rate of soil respiration, high Eh condition and enhance input of atmospheric CO_2_. Soil carbonates analyzed for other time period fall within a wide range of δ^13^C values covering entire year with high and low soil respiration rate, variable Eh and atmospheric CO_2_ input conditions. Furthermore, the clumped isotope temperature estimates for soil carbonates support the high CO_2_ level predicted earlier using δ^13^C values of soil carbonate and their organic matters^8^.

### Latitudinal position of the Indian plate and Uncertainty

**Figure 2:**
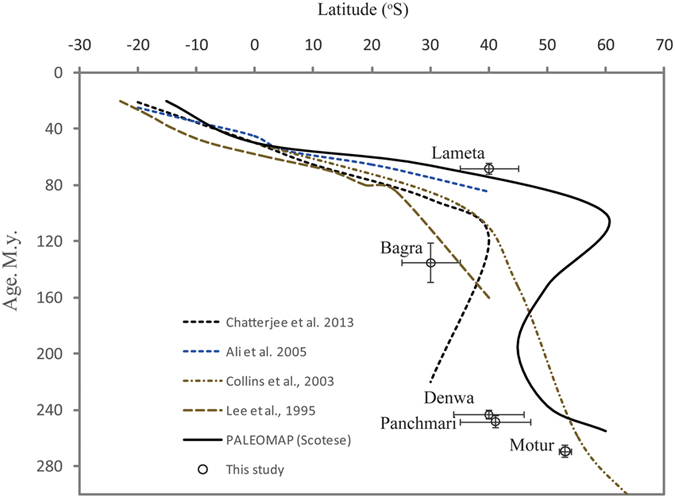
Position of the Indian plate during the Phanerozoic period is shown here while it drifted from the southern hemisphere to its present position in the northern latitude. The exact position and uncertainties of plate location are based on the palaeo-magnetic data^1^,^2^,^3^,^4^,^31^. The palaeosol samples used in this investigation were dated based on the presence of vertebrate fossils^10^,^11^,^15^. A paleo-latitudinal reconstruction from specific soil carbonates isotopic data involves use of empirical relationship between δ^18^O of meteoric water (GNIP data) and latitudes (see Extended Data Fig. 1). The δ^18^O of past meteoric water was estimated after taking into account evaporative fractionation at different latitudes.

The available paleogeographic reconstructions suggest that the Satpura Gondwana basin was positioned at 60°S in the Early Permian and moved to 30–40°S by the Middle Triassic^30^,^31^,^32^ and thus traversed through different climatic zones during this period. Existing paleomagnetic data suggest the position of the Indian plate relative to its current position (Fig. 2). Accurate positioning of the Indian plate during Phanerozoic is based upon a variety of archives; including sea-floor magnetic anomalies, geochronology and palaeo-magnetic data on flood basalt and relative position of hot spot track^33^. At present, knowledge the exact positions (with uncertainties) of Indian plate are based on the compilation of data^30^,^31^ from various sources including the on-going PALEOMAP (http://www.scotese.com/) project. The palaeo-latitude of India is determined from global synthetic apparent polar wander paths (APWPs) which place the central Indian region at 53°S during the deposition of the Motur sediments^30^,^31^. The average position of the Indian plate during the Triassic period was 44°S and shifted to nearly 35°S by the late Jurassic period. Finally, the Indian plate was located at 22°S during the Cretaceous period. There are several reasons not to rely on the global APWP. First, their curve extends back only to 200 M.y., whereas we are interested in motions back to the Permian. A late Permian and Triassic segment to the Besse and Courtillot^34^ reconstruction of plate movement was found to be geologically implausible and an alternative approach to validate the position of Indian plate is in high demand^35^.

This use of stable isotope in terrestrial carbonate for predicting latitude has been successfully demonstrated in reconstructing the locations of continents in the northern hemisphere^5^,^7^,^36^. However, in majority of these studies the predicted value of δ^18^O in calcite as a function of latitude were likely compromised by absence of information about soil temperature, depth of soil calcite precipitation, and evaporation of soil water. Here we obtained soil temperature from Δ_47_ and accommodated evaporation effect. Effect of elevation governing the isotopic composition of precipitation was considered minimal as the palaeo-elevation of the basins is unknown. However, based on sedimentary record documenting structures associated with the strata where soil carbonates were deposited it is concluded that most of the deposition was confined to a flood plain setting at elevation close to mean sea level^9^,^10^,^11^. The δ^18^O_smow_ values of water in equilibrium with soil carbonates varies through the section and averages −8.9‰ in the Motur Formation, −4.3‰ in the Pachmarhi Formation, −4.0‰ in the Denwa Formation, 0‰ in the Bagra Formation, and −3.4‰ in the Lameta Formation. Allowing corrections for the soil water δ^18^O_smow_ values due to the effect of processes like evaporation. We translated the soil water δ^18^O_smow_ to true meteoric water δ^18^O_smow_ values^5^ by including a constant evaporative enrichment factor of 2‰ for Motur carbonates and 2.5% for rest of the carbonates from four stratigraphic levels (See Supplementary data). The soil water isotopic values resemble values for the meteoric water after correction and thus been suitably used as input values in the empirical model for predicting the latitudinal position (Extended Data Fig. 1) of Indian plate. However, uncertainties on the calculated δ^18^Ow due to error in determination of isotopic fractionation factor between water and calcite was ignored. The latitudinal position varied during the deposition of sediments across the succession and the average position are 53 ± 1°S during deposition of Motur sediments, 41 ± 6°S for the Pachmarhi sediments, 40 ± 6°S for the Denwa sediments, and 30 ± 5°S and 40 ± 5°S for the Bagra and Lameta Formations respectively. The approach is validated for the modern samples of soil carbonates from India^28^ which yielded latitude position of 34 ± 1°N as compared to the actual position of 27–31°N latitude (Table 1). Uncertainties in our estimates are within 20% of the true value (as explained in the Extended Data Fig. 2) for samples lying within 30°S latitude. Our results are consistent with previous paleo-latitudinal estimates but provide new constrains on the position in cases where limited data of APW is retrieved from the sedimentary sequences. The uncertainties in the paleo-latitudinal estimates are mainly due to the factors like scatter in the Δ_47_ values and δ^18^O of carbonates. Soil carbonates of the Lameta, and Bagra formations, being formed close to the mid latitudes, yielded large uncertainties while carbonates deposited at low latitudes, namely in the Denwa, Panchmari and Motur Formations are less uncertain in terms of their latitudinal position.

### Hydrological circulation during periods of elevated greenhouse gases level

**Figure 3:**
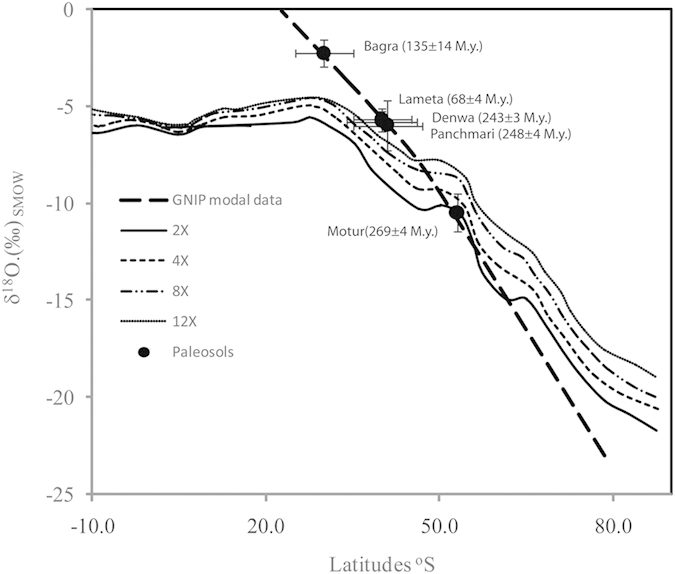
Reconstruction of paleo-hydrology using analyzed clumped isotope ratios in palaeosol carbonates and δ^18^O of carbonates. The values are compared with the GNIP observation and model based estimates of meteoric water isotopic composition at varying level (2x, 4X, 8X and 12X) of atmospheric CO_2_. The vertical error bar denotes the δ^18^O of precipitation estimates established analyzing number of samples of paleosol carbonates from specific startigraphic horizon as described in the text. A large offset from the model results was noted for the carbonates from Bagra formation, suggesting anomalously excess evaporation during the deposition of these sediments.

Ghosh *et al*.^8^ previously used the carbon isotopic composition of inorganic and organic remnants from the same set of palaeosol carbonates to derive *p*CO_2_ in the Phanerozoic period. Our previous estimates showed that the CO_2_ content of the atmosphere during depositional of the Motur formation was 540–890 ppmv while the concentration increased to 910–1510 ppmv during the period of deposition of soil carbonate in the Denwa formation. Similarly, the *p*CO_2_ content registered analyzing the samples from Bagra formation was 1675–2775 ppmv, while *p*CO_2_ content of atmosphere recorded analyzing the Lameta samples was 1110–1850 ppmv. These estimates are in agreement with the GEOCARB model prediction^37^. In this study, we determine the temperature of carbonate precipitation and obtain more precise estimates of the isotopic compositions of ancient soil waters and precipitation. The estimated water isotopic compositions were used for predicting the latitudinal position for the carbonate depositions and compared with the modal GNIP data for the prediction (Fig. 3). We compare our soil water compositions with precipitation isotopic compositions simulated using the GENESIS GCM for a range of elevated atmospheric CO_2_ levels^24^ (Fig. 3). The meridional variability of soil water isotopic compositions compares reasonably well with the simulated meridional variability in zonal-average precipitation, indicating that to first order ancient soil water does track paleo-latitude. The comparison also demonstrates a systematic isotopic enrichment of most meteoric waters relative to both modern observed and simulated precipitation. The meteoric δ^18^O values recorded in the carbonates during 269 M.y. until 135 M.y. are enriched compared to modern day average isotopic composition of precipitation across the latitude and even exceeded the simulation trend at higher *p*CO_2_ condition.

## Conclusion

The paleosol carbonates were precipitated in fluvial sediments during Permian to Cretaceous periods, coinciding with the movement of Indian landmass towards north and is overlain by the Deccan basalt on the top. The stratigraphic position of the palaeosol carbonates restricted water rock interaction and late diagenetic transformation of the original signatures. Clumped isotopic analysis on these samples reveals the temperature of calcite precipitation and the oxygen isotope ratios of carbonates. We note that the clumped isotope temperatures are lower than GCM-simulated annual surface temperatures, likely indicating a seasonal bias in carbonate precipitation. The isotopes in precipitation show very good agreement with the simulated meridional distribution. Not only does this validate using soil water δ^18^O as a paleo-latitudinal proxy, it also implies that the global-scale hydrological cycle was not fundamentally different during the climates of the late Paleozoic and Mesozoic. The δ^18^O values derived here are more robust than the temperature values as it captured either environmental water at the time of calcite precipitation or later during the process of early diagenesis. The secondary calcites precipitated from the diagenetic water at higher temperature closely resemble the composition of environmental water. This suggests that the early diagenetic water is similar to the environmental water. This is documented in our observation. The temperature trends recorded in the sample are consistent with the model-based estimates, except for the 269 M.a. samples, which is much too warm. Also, pedogenic carbonates have a tendency of becoming increasingly enriched in δ^18^O at higher MAT and evaporation rates^3^,^32^. A systematic enrichment of 0.5‰ would bring all the points in line with the model predictions. Therefore, any offset in paleo-latitude reconstruction can partly be explained by adopting a systematic isotopic enrichment^7^. The approach of using the latitudinal variability of oxygen isotopic composition in rainfall is used here to estimate the position of Indian plate and shows great promise, especially at mid and high latitudes where isotopic gradients are greatest. This method could be further refined by simulating specific time slices in the past and comparing carbonate δ^18^O with simulated δ^18^O at specific locations. Based on the data we observe variations in the rate of drifting of Indian plate. Assuming the orientation of Indian plate remaining unchanged during its journey to the north, we can deduce the rate of movement of Indian plate with shift in the mean latitudinal position with age. Assuming 111 km correspond to a drift of 1° latitude, we estimate the rate of migration was 51 mm/year between 269 M.y. and 243 M.y., dropping to 10 mm/year during the interval between 243 M.y. ago and 135 M.y ago.

## Additional Information

**How to cite this article**: Ghosh, P. *et al*. Tracking the migration of the Indian continent using the carbonate clumped isotope technique on Phanerozoic soil carbonates. *Sci. Rep.* **6**, 22187; doi: 10.1038/srep22187 (2016).

## Supplementary Material

Supplementary Information

## Figures and Tables

**Table 1:** Stable isotope and clumped isotopic composition of Satpura palaeosol carbonates with details about their stratigraphy, temperature.

Age. M. y.	Age. M. y.	**Δ_47_(‰)	T (°C)^e^	δ^13^C(‰) _VPDB_	δ^18^O(‰)_VPDB_	δ^18^O_precip._(‰)^d^^+^^c^^SMOW^	MAAT^b^	Latt. Est^a^
Modern India*		0.66 ± 0.01	36.2 ± 2(7)	−1.6	−5.8	−3.7	22	34° N ± 1
Lameta	68 ± 4	0.65 ± 0.04	39.0 ± 8(13)	−10.0	−8.2	−5.7	25	40° S ± 5*
Bagra	135 ± 14	0.67 ± 0.10	39.7 ± 6(8)	−6.4	−5.0	−2.3	26	30° S ± 5
Denwa	243 ± 3	0.71 ± 0.04	25.2 ± 8(13)	−8.1	−5.7	−5.8	8	40° S ± 6
Panchmari	248 ± 4	0.65 ± 0.12	20.3 ± 7(6)	−8.7	−4.9	−6.0	3	41° S ± 6
Motur	269 ± 4	0.62 ± 0.09	34.1 ± 2(4)	−6.8	−12.6	−10.5	19	53° S ± 1

^**^Δ_47_ calibrated in CDES scale correction (Yoshida *et al*., 2013).

^a^Equation LAT (°S) = 0.038 * (δ^18^O precip.)^2^ + 3.319* δ^18^O precip- 22.35 (see Supplementary) estimates and deduction of Latitudinal position.

^b^Estimation of mean air temperature (Quade *et al*.^28^) MAAT (°C) = 1.2 × (T°C Δ_47_)-21.72 (r^2^ = 0.92).

^c^Inorganic calcite precipitation fractionation equation Kim and O’Neil^15^ 1000lnα = 18.03(1000/T)-31.82.

^d^Evaporation effect to modify the soil water composition (2.5‰ for samples lying between 45–30°S while 2% for samples lying beyond 50°S).

^e^Temperature estimation Δ_47_ = 0.0636 ± 0.0049 × 10^6^/T^2^−0.0047 Dennis *et al*.^26^.
