# Supplementary material for: Tracking the migration of the Indian continent using the carbonate clumped isotope technique on Phanerozoic soil carbonates
Source: Sci Rep. 2016 Mar 2;6:22187. doi: 10.1038/srep22187 (PMC4773985; doi:10.1038/srep22187)
Supplement: Supplementary Information [file srep22187-s1.doc]

**Supplementary extended data**

**Tracking migration of Indian continent using clumped isotope technique in Phanerozoic soil carbonates**

### Prosenjit Ghosh, Mikhail V. Vasiliev, Parthasarathi Ghosh, Soumen Sarkar, Sampa Ghosh, Keita Yamada, [Yuichiro Ueno](https://www.google.co.in/url?sa=t&rct=j&q=&esrc=s&source=web&cd=2&cad=rja&uact=8&ved=0CCEQFjAB&url=http%3A%2F%2Fwww.geo.titech.ac.jp%2Flab%2Fueno%2F&ei=-NSvVM7QBsaPuATtqoLICw&usg=AFQjCNEiVAWPz_4rA6AsVGDE6qG1BsZwFg&sig2=c1HDeYgV_giSSo_bmYdS0w&bvm=bv.83339334,d.c2E), NaohiroYoshida, Christopher J. Poulsen

**Methodology**

The carbonate “clumped-isotope” thermometer is based on the abundances in carbonate minerals of carbonate ion groups that contain both the ^13^C and^18^O atoms (i.e., ^13^C^18^O^16^O_2_)^14^.These ionic groups have lower zero point energies than their isotopically ‘normal’ and singly‐substituted relatives (i.e., ^12^C^16^O_3_, ^13^C^16^O_3_, ^12^C^17^O^16^O_2_ and ^12^C^18^O^16^O_2_), leading to a thermodynamic driving force that promotes ‘clumping’ of rare isotopes into bonds with each other as opposed to being randomly dispersed throughout the mineral lattice^14,39^. This effect can be described using an isotope exchange reaction among isotopologues of the carbonate ion^40^:

^12^C^18^O^16^O_2_ + ^13^C^16^O_3_ ↔ ^13^C^18^O^16^O_2_ + ^12^C^16^O_3_ (1)

The equilibrium constant for this reaction increases with decreasing temperature, and can be determined by digesting a carbonate mineral in phosphoric acid and measuring the δ^18^O, δ^13^C, and abundance of mass‐47 isotopologues (mostly ^13^C^18^O^16^O) in product CO_2_. From these data, one can calculate the enrichment of mass‐47 isotopologues in product CO_2_ relative to the ‘stochastic’, or random, distribution of all C and O isotopes among all possible isotopologues^41^. This enrichment, termed the Δ_47_ value, is proportional to the equilibrium constant for reaction 1 in reactant carbonate and varies with carbonate growth temperature by the relationship for the first time proposed by Ghosh et al. (2006)^14^:


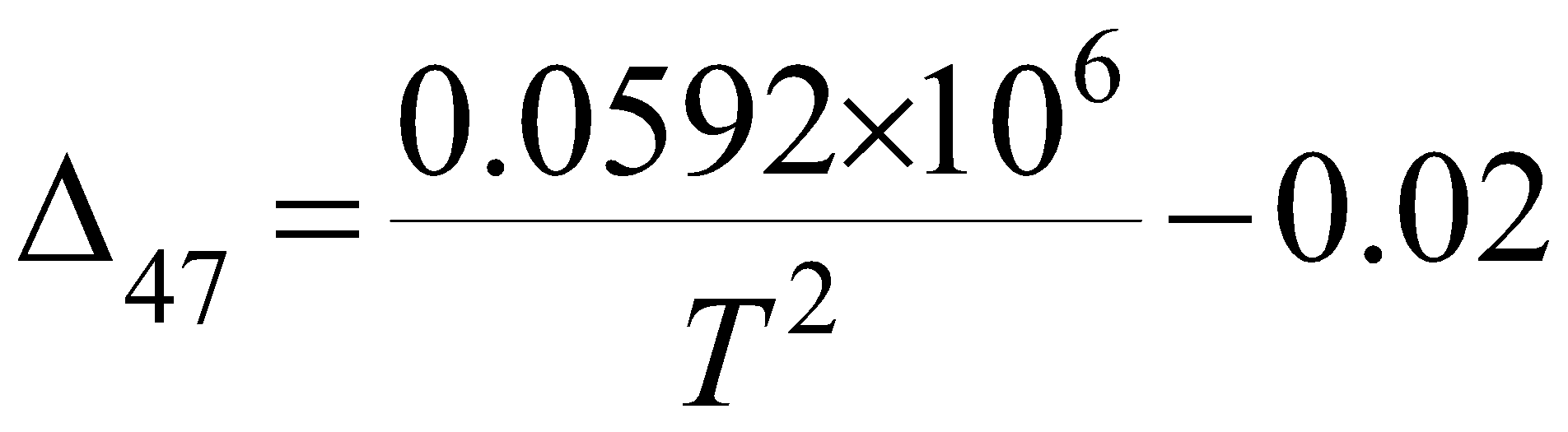


(2)

where Δ_47_ is in units of per mil and T is temperature in Kelvin. Later modified as Δ_47_=0.0636±0.0049 x 10^6^/T^2^-0.0047 in a Absolute reference frame^26^. Standard errors in Δ_47_ are on the order of ±0.01‰, leading to errors in temperature of calcite 2 °C. The key feature of this thermometer is based on a homogeneous equilibrium within carbonate rather than on a heterogeneous equilibrium between carbonate and water or some other phase. Therefore, unlike conventional carbonate-water oxygen isotope thermometry, it does not require knowing the isotopic composition of water from which the carbonate grew^14, 39-42^.

***Experimental protocol***

The experimental procedure of sample preparation for “clumped-isotope” analyses is described by McCrea and Swart et al^42,43^. All the carbonate samples analyzed at the Tokyo Institute of Technology, Japan, methodology and analytical protocol are described in complete detail in Yoshida et al^44^.

For each analysis, approximately 50 mg of the carbonate powder and 1 ml of phosphoric acid were transferred into the two arms of a McCrea type reaction vessel. This was evacuated at a pressure of 10^-4^ m bar level using combination of Turbo and rough pump. The complete setup was disconnected and kept inside a water bath maintained at a constant temperature of 25±0.5 ^o^C for the reaction. The CO_2_ generated during reaction was extracted following the standard protocol^44^. Purification steps involved removal of water vapour and other contaminants which was achieved by combination of liquid nitrogen trap and a mixture of dry ice and ethanol slush. The CO_2_ once extracted in a cold finger was entrained with helium stream through a capillary column (PoraPLOTQ, 25 m x 0.32 mm i.d.; Varian Inc., Palo Alto, CA, USA) to a gas chromatograph (HP-6890; Agilent Technologies Inc., Santa Clara, CA, USA) which was held at -10^o^C for separation of CO_2_ from other trace hydrocarbon and halocarbon. Eventually, the purified CO_2_ sample condensed into a glass cold finger and was analyzed using the IRMS.

Analytical measurement was done for δ^13^C, δ^18^O and mass-47 isotopologues of CO_2_ on isotope ratio mass spectrometer (ThermoFinniganDELTA^plus^XP IRMS) and configured to collect masses 44, 45, and 46 (read through 10^8^ to 10^11^ ohm resistors), as well as 47, 48, and 49 (read through 10^12^ ohm resistors) simultaneously. The 48 and 49 masses were also performed in order to ensure no isobaric interferences due to the presence of contaminants. These measurements were done in a dual inlet mode with a typical source pressure, sufficient to maintain the mass-44 ion beam at a voltage of 10 – 12 V. Each analysis involved six acquisition lines with 10 cycles in it with a signal integration time of 8 s per measurement. The reference CO_2_ gas (Oztech Trading Corp., Safford, AZ, USA) has δ^13^C and δ^18^O values of –10.97 ‰ (w.r.t. VPDB) and +31.31‰ (w.r.t. VSMOW), respectively established based on analysis of NBS-19 international carbonate standard. The analyses were standardized using NBS-19, heated gas and in-house Wako synthetic calcium carbonate (Wako Pure Chemical Industries Ltd., Osaka, Japan; hereinafter Wako). Calibration of the Wako CaCO_3_, which was ran more frequently during the course of our measurement was done by adopting two published values of reference materials i.e. NBS-19 and heated CO_2_ at 1000°C. Absolute reference frame adopted the Δ_47_ value of 0.392 for NBS-19 and for heated CO_2_, the value of 0.0266 was accepted based on theoretical estimates by Wang et al. (2004)^46^. The long-term reproducibility of NBS-19 and in-house Wako standards is described in the standardization paper from Tokyo Institute of Technology^44^. For the period 2007–2011 NBS-19 (n=12) yielded values for Δ_47_ is 0.330 ± 0.019 (1SD) in heated gas scale. Similarly, for Wako reference material (n=59) analysed during the same time span yielded Δ_47_ value of 0.639 ± 0.021 (1SD) respectively^44^. All the palaeosol samples were analyzed during the period 2007 till 2009.

In order to convert our D_47_ value to Δ_47-HG_, in a few cases CO_2_ sample after analyses was recovered in transferred to a ultrapure synthetic quartz tube (6 mm o.d. provided by Shin-Etsu Chemicals Co. Ltd., Tokyo, Japan) and sealed after evacuation. The sealed tube containing CO_2_ sample was heated in a muffle furnace at 1000^o^ C for 1-2 hours and then quickly quenched to room temperature. This difference between the D_47_ values measured in the samples CO_2_ and heated CO_2_ D_47_ values at temperature 1000 ^o^C allowed establishing the

Δ_47-HG_ value in heated gas scale^14^. All data are reported at the 25^o^C temperature reaction and therefore no additional correction for the reaction temperature fractionation factor was applied. For 2007 – 2011 years, heated CO_2_ yielded D_47_ (n=114) values of –1.074 ± 0.043 (1SD, ‰)^44^. The values in the heated gas scale are converted to absolute scale by using the equation 6 in Yoshida et al., (2013)^44^ paper and is given here as:


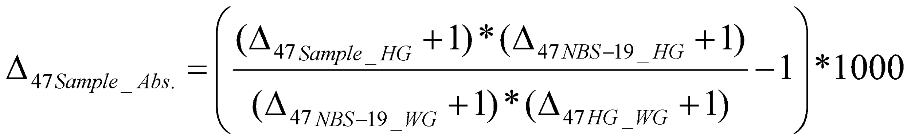


(3)

where the subscripts ’NBS_HG’, ’NBS_WG’ and ’HG_WG’ represent theΔ_47_ value of sample with respect to the stochastic distribution, the NBS-19 value with respect to heated CO_2_, the NBS-19 value with respect to Oztech reference CO_2_ or working gas and the heated CO_2_ value with respect to Oztech reference CO_2_ or working gas respectively.

**The Δ_47_, δ^13^C and δ^18^O data**

Carbonate powders from 54 vertisols collected across the entire stratigraphy of Satpura basin were analyzed for Δ_47_, δ^13^C and δ^18^O. The average Δ_47_, δ^13^C and δ^18^O values with their standard deviation corresponding to all the samples from each formation with stratigraphic ages were used for interpretation of environmental condition (Table 1, Figure 1). The Δ_47_ values along with uncertainty i.e. 0.617 ± 0.085 (1SD, ‰, n=6), 0.645 ± 0.127 (1SD, ‰, n = 10), 0.711 ± 0.040 (1SD, ‰, n = 13), 0.666 ± 0.102 (1SD, ‰, n = 12) and 0.648 ± 0.039 (1SD, ‰, n = 13) for the Motur, Pachmarhi, Denwa, Bagra and Lameta formations respectively are displayed in the Table 1, Figure 1. Further, in order to estimate the temperature the revised relationship between Δ_47_ –T in absolute reference was used:

Δ_47_ = (0.0636 ± 0.0049
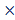
 10^6^)/T^2^ – (0.0047 ± 0.0520) (calibration range 1–50^o^C) (4)

Figure. 1, (d) shows a calcification temperature calculated from the Δ_47_ values using the Δ_47_–T dependency equation given above by Ghosh et al., (2006)^14^ and later revised by Dennis at al. (2011)^26^. The temperature values obtained from the revised thermometry of Zaarur et al., (2013)^27^ is near identical and will cause no major effect on the overall change in the estimates of latitudes. Therefore, we used original Ghosh et al., (2006)^14^ which was later confirmed for soil carbonates temperature calibration by Quade et al., (2013)^28^, where MAAT estimates were related with the effective temperature derived from the Dennis et al., (2011)^26^ relationship. The carbon isotopic composition varied between -6.64 ± 0.86(1 SD ‰, n= 12) to -12.52 ± 1.79 ‰ with samples belonging to younger age registering lighter values, similarly δ^18^O showed ranges between -5.52 ± 2.20 ‰ to -11.6 ± 2.0 ‰, with older samples recording values which are more lighter. These values were consistent with previous observation from separate set of samples from each strata^8^.

**Deducing the Latitudinal position**

A first step toward understanding the position of carbonate deposition in spatial domain is accomplished through identification of δ^18^O and temperature signature by analyzing the carbonates and then translates that to δ^18^Oppt. This involves number of steps which are introduced to get more accurate idea of δ^18^Oppt. Studies have shown that soil carbonate inherit the isotopic signature of local meteoric water. Model of predicting δ^18^O of soil carbonate as a function of latitude on the modern earth incorporates three of the most important variables affecting the oxygen isotopic composition of soil calcite: the isotopic composition of meteoric water, the ambient soil temperature at the depth of calcite precipitation, and evaporation of soil water^5^. The relationship between δ^18^O of meteoric water and latitude is based on the data compilation of GNIP data available from the 37 stations scattered across the southern hemisphere for generating a model which is similar to a model already been proposed for the northern hemisphere (Extended Data Figure 1)^36,45^. The relationship between δ^18^O of meteoric water and latitude is strongest at mid to high latitudes and the model presented here is restricted to latitudes between 20°and 60°. The equation which represents best fit of data points is:


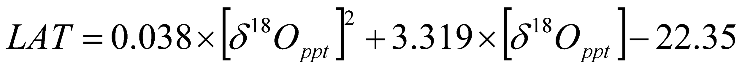


……………… .(5)

Quade et al., (2013)^28^ analysed modern day soil carbonates from Northern hemisphere where isotopic composition of δ^18^O of meteoric water was deduced following the steps included in our method for palaeosol carbonates. The latitude position of India matches with the observed location documented in their study. Equation (5) was applied to each GNIP station, and a second set of residuals was calculated that describes the difference between observed values and those predicted by the model (Extended data Figure 2). Regions of high-magnitude residuals (plotted as % deviation from the observed values) occur at middle to high latitudes (20-30°S) and likely shows effect of zonal heterogeneity of moisture source and long distance vapor transport.

**^13^C-^18^O bond in past soil carbonates**

The traditional oxygen isotopic compositions of the calcite cements exhibit a negative correlation with the latitudinal position^8^; however, in the absence of information on precipitation temperature, it was difficult to exclude the possibility of a temperature effect on isotopic variability. Here, we used a clumped isotope values from calcites to retrieve the temperature for calcite precipitation. These data were also compared with previous data from Ghosh et al. (2001)^8^. Significant discrepancies were discovered in δ^13^C for the Lameta formation (68 ± 4M. y.). Such variability might possibly be explained due to the presence of clay bed, pebbly sandstones and calcic vertisols recording variable diagenetic modification (Extended Data Figure. 3)^11^. Further, we calculated the δ^18^O values of waters in equilibrium with the calcite. The Δ_47_ values of a few samples, however, do appear to have been affected to some degree during subsequent burial diagenesis. Additionally, to show the relationship between δ^13^C and δ^18^O data of all the samples of pedogenic carbonates were plotted using Extended Data Figure 3. Samples showing signature of significant deviation from the mean value (±2σ from the mean) were designated as outliers. The pedogenic carbonates from the Motur formation with assigned age of 269±4 M.y. yielded an average value of 0.62 (±0.085, n=4) ‰ (excluding two outliers at 0.48 and 0.54 which, we suspect, reflect a high temperature diagenetic resetting), similarly, pedogenic carbonates belonging to the Pachmarhi formation deposited during 248±4 M.y. yielded an Δ_47_ value of 0.65 (±0.12, n=6), while sample analysed from the Denwa formation, deposited during 243±3 M.y. yielded an Δ_47_ value of 0.71 (±0.040, n=13) ‰ (excluding three outliers that, we suspect, reflect a diagenetic resetting; see footnote 10), well preserved soil carbonate found in the Bagra formation deposited during 135±14 M.y yielded an Δ_47_ value of 0.67 (±0.1, n=9) ‰ (excluding two outliers that, we suspect, reflect a diagenetic resetting) and those from the Lameta formation belonging to the age of 68±4 M.y. yielded CO_2_ with an Δ_47_ of 0.65 (±0.04, n=13) ‰ (Table 1 ). Note that the samples population belonging to the age of 248 M.y. and 243 M.y. are indistinguishable and can form a single group without substantial deviation in the average values. The apparent temperatures of carbonate growth based on clumped isotope thermometry and the calculated δ^18^O values of soil water under equilibrium condition are presented in Table 1. Sample originating from Lameta and Bagra formation are less altered as seen from the clustering of δ^13^C and δ^18^O values in the scatter plot. Carbonates from Pachmarhi formation showed large variability in carbon and oxygen isotopic composition probably suggesting seasonal precipitates. Palaeosol carbonates analysed from Denwa and Motur formation are correlated in terms of δ^13^C and δ^18^O values, probably suggesting shallow level precipitation of carbonate in a soil horizon or carbonates growth during seasonal dry and wet conditions^9^.

**^13^C-^18^O reordering of carbonate**

Although the entire stratigraphy is 5 km thick but bulk of the Satpura succession was deposited in a mega half-graben bounded by basin margin faults and sediment accumulation took place under different fault-controlled subsidence regimes with intervening tectonically static periods. Also, the subsidence rate varied across the basin resulting in an asymmetric basin fill with the thickness increasing towards the north^10^. Estimated maximum burial depth for all samples is less than 900 m^10,12^. The burial depth is translated into maximum burial temperature of ~30 °C (assuming a 30 °C/km geothermal gradient), well below the proposed temperature of ~100–200 °C above which self-diffusion can alter ^13^C-^18^O bonding distribution in carbonate lattice^26,38^. Finally, the Δ_47_ temperature, δ^18^O, and δ^18^O_water_ data confirm no deep burial diagenesis or late diagenesis for majority of the samples analysed from Motur, Denwa and Panchmari formation, while samples from Bagra and Lameta formation experienced ^13^C-^18^O reordering to some extent as displayed in a plot of Δ_47_ temperature versus depth (Extended Data Figure. 3). Samples with lower values of temperatures were found well preserved and reliable in term of δ^18^O_soilwater_ reconstruction.

**Estimation of Mean annual air temperature**

Further, we estimated the mean annual air temperature (MAAT) from the soil temperature data after applying the proposed offset correction recommended in Quade et al., (2013)^28^; where MAAT =1.2 (T_eff_)-21.72, where T_eff_ is effective temperature required to account for the Δ_47_ variability at each startigraphy, after consideration of soil depth and ground heating (Extended data Figure 4). The observed pattern showed low temperature estimates for Denwa and Panchmari samples and higher estimates for Motur samples. Inconsistency in predicted MAAT with pCO_2_ record available from the past suggests following possibilities: fallacy of implementing the proposed correction equation^28^ for soil carbonate deposited in the Southern hemisphere and improper knowledge of soil depth during soil carbonate deposition in the geological past. However, effective soil temperature data shows expected trend of high temperature at high pCO_2_ levels known so far based on carbon isotope study in the soil carbonates.

**References**

39. Schauble, E. A., Ghosh, P. & Eiler, J. M. Preferential formation of ^13^C–^18^O bonds in carbonate minerals, estimated using first-principles lattice dynamics. *Geochim. Cosmochim. Acta***70,** 2510–2529 (2006).

40. Affek, H. P. Clumped isotope palaeothermometry: Principles, applications, and challenges. *Paleontological Society Papers* **18.** 101–114 (2012).

41. Huntington, K. et al. Methods and limitations of 'clumped' CO_2_ isotope (Δ_47_) analysis by gas-source isotope ratio mass spectrometry, *J. Mass. Spectrom.* **44,** 1318–1329 (2009).

42. McCrea, J. M. On the isotopic chemistry of carbonates and a paleotemperature scale. *J. Chem. Phys.* **18,** 849–857 (1950).

43. Swart, Pn. K., Burns, S. J. & Leder, J. J. Fractionation of the stable isotopes of oxygen and carbon in carbon dioxide during the reaction of calcite with phosphoric acid as a function of temperature and technique. *Chemical Geology: Isotope Geoscience section* **86,** 89–96 (1991).

44. Yoshida, N. et al. Precision and long-term stability of clumped-isotope analysis of CO_2_ using a small-sector isotope ration mass spectrometer. *Rapid Commun. Mass Spectrom.* **27,** 207–215 (2013).

45. Fricke, H. C. & O’Neil, J. R. The correlation between ^18^O/^16^O ratios of meteoric water and surface temperature: its use in investigating terrestrial climate change over geologic time. *Earth and Planetary Science Letters* **170,** 181–196 (1999).

46. Wang, Z., Schauble, E. A. & Eiler, J. M. Equilibrium thermodynamics of multiply substituted isotopologues of molecular gases. *Geochim. Cosmochim. Acta* **68,**

4779–4797 (2004).

**Extended data and Figures**


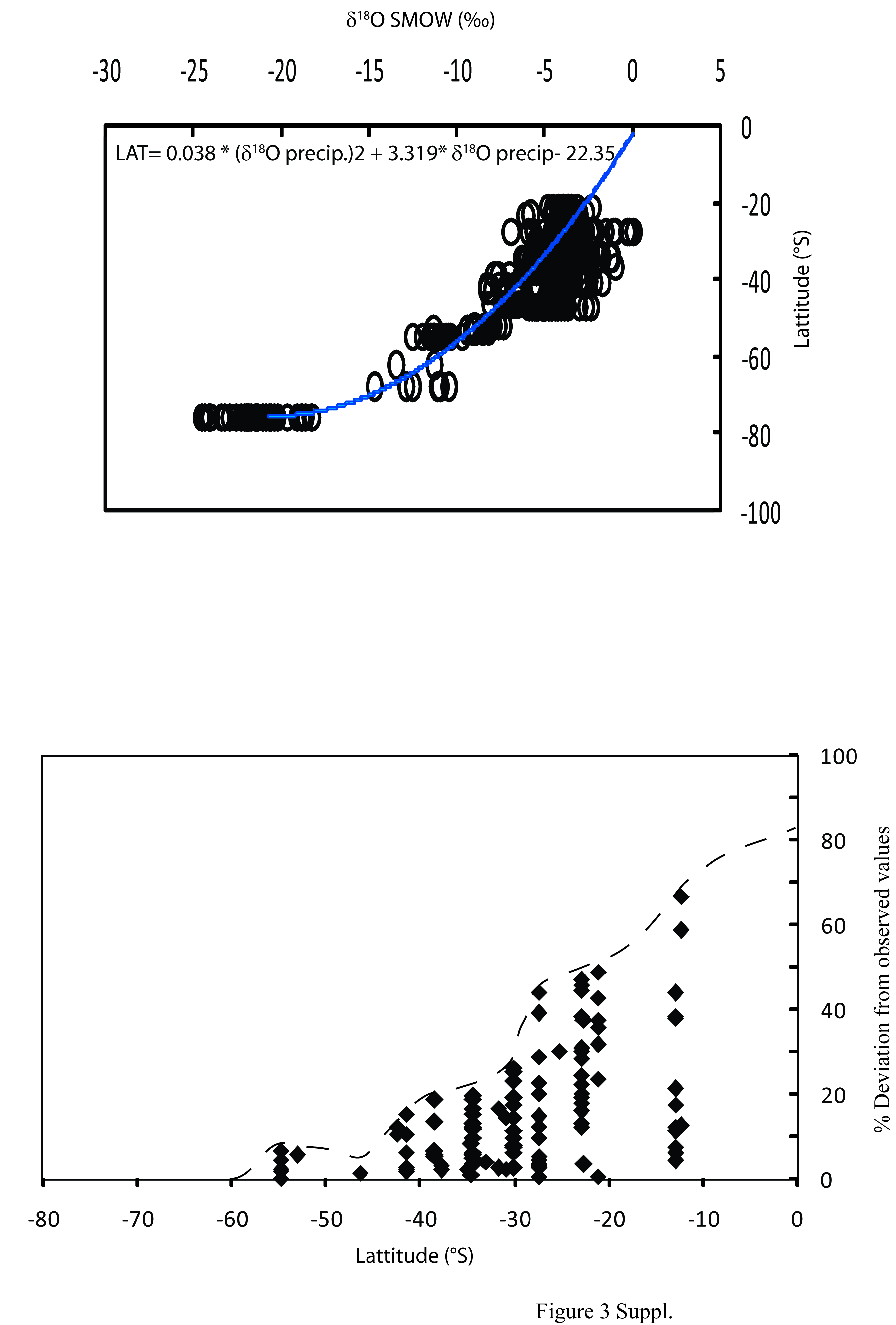


**Extended Data Figure 1:** δ^18^O of meteoric precipitation plotted against station latitude for GNIP stations in the southern hemisphere, with best-fit polynomial for model equation for deducing the palaeo latitudinal position. Note that the data points are close to the best fit polynomial line until 20°S latitude.


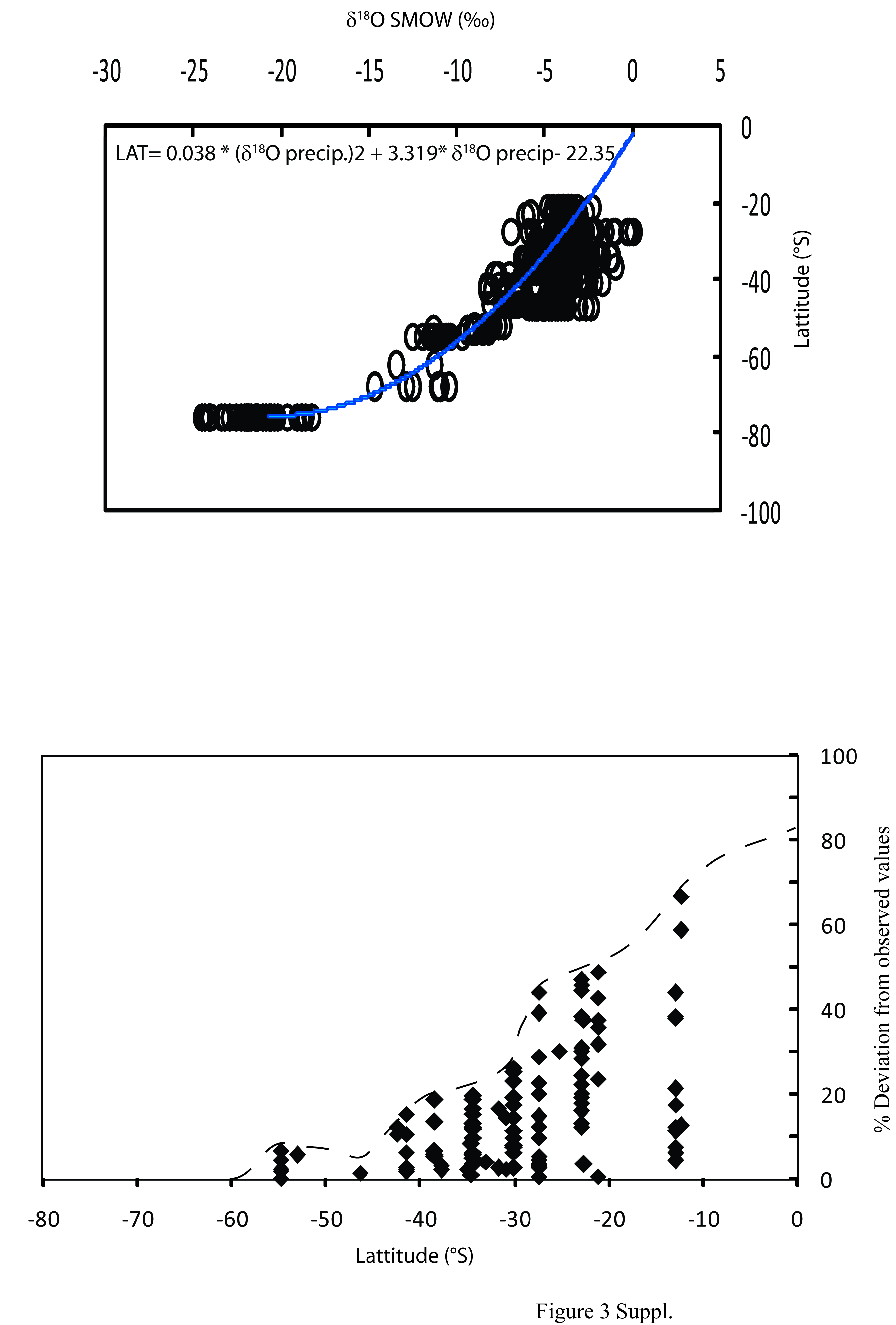


**Extended Data Figure 2:**

Deviation of predicted values from the observation using the latitudinal reconstruction equation where δ^18^O of meteoric water is input parameter. Note that the predicted values are well within 50% from the observed till 20°S latitudes. However, predicted values are within 20% of the observed values till 30°S.


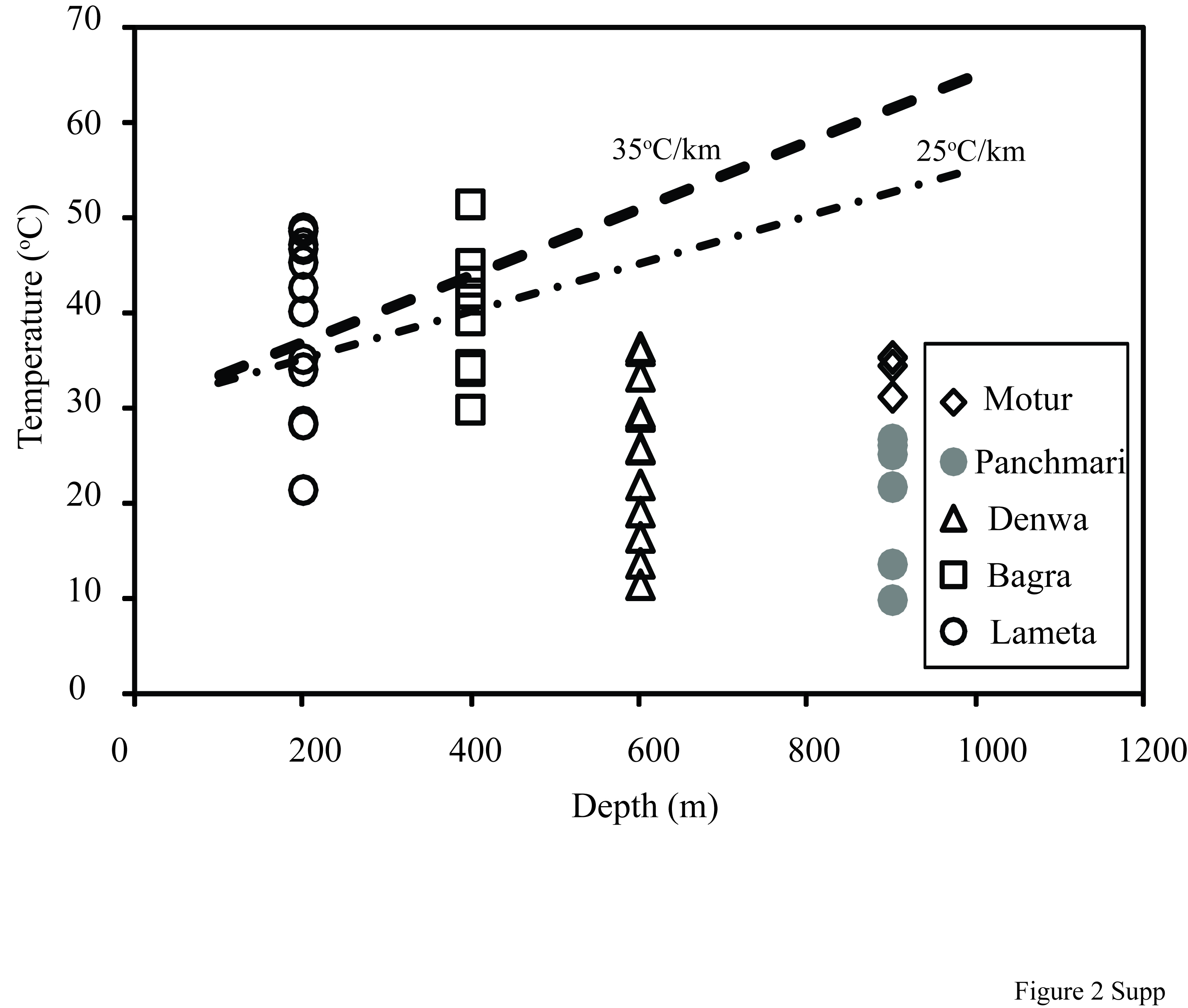


**Extended Data Figure 3**: Temperature from clumped isotope thermometry and modelled

Maximum burial temperatures (dashed lines) plotted as a function of burial depth. Maxium depths of burials for carbonates from different lithostratgraphic unit are obtained from Ghosh et al., (2012). The burial temperatures are modeled for 35 °C/km and 25 °C/km geothermal gradients. Evidence of ^13^C-^18^O reordering in the palaeosol carbonates resulting from diagenetic processes at depth (for example, dissolution/re crystallization, pressure solution, or other mechanisms of crystal coarsening) is absent.


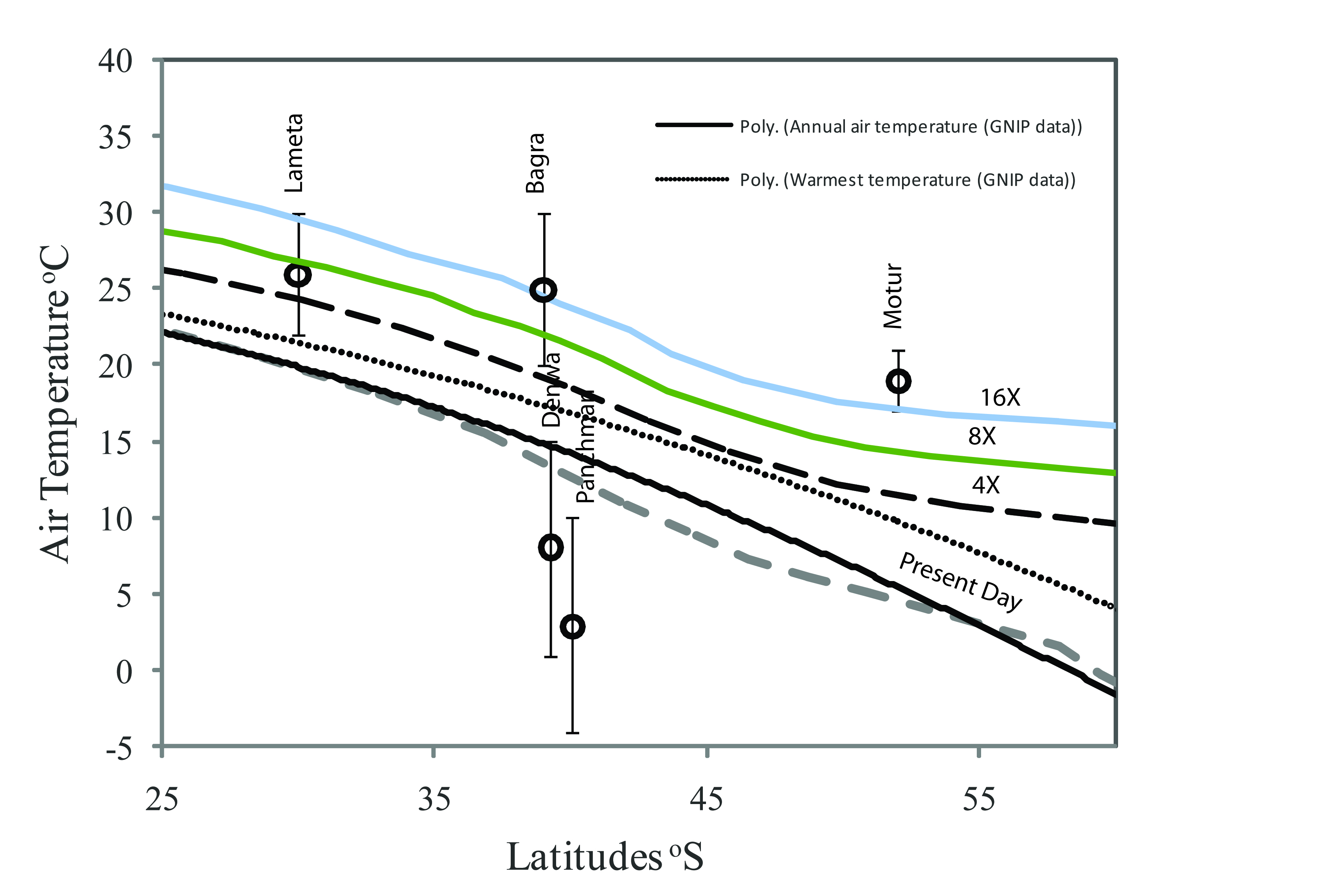


**Extended Data Figure 4:** Estimated Mean annual air temperature from the clumped isotope data after correcting for the offset in soil temperature as prescribed in Quade et al., (2013)^28^ .

| Supplementary Table 1. Stable isotopic composition of Satpura paleosol with details about their stratigraphy and nature of samples. Also displayed in this temperature estimates using Ghosh et al., (2006) and revised thermometry equation proposed by Zarrur et al., (2013) equation. Other detail includes mass 48 and 49 in the sample and Estiamtes of water composition using Kin and O'Neil (1997) equation. | | | | | | | | | | | | |
| --- | --- | --- | --- | --- | --- | --- | --- | --- | --- | --- | --- | --- |
|
|
|  |  |  |  |  |  |  |  |  |  |  |  |  |
| Age. M. y. | Sample | *Δ_47_(‰) | stder (n=6) | stdev | **Δ_47_(‰) | Δ_48_(‰) | Δ_49/44_(‰) | `T.^o^C | δ^13^C(‰)VPDB | δ^18^O(‰)VPDB | Date of collection | Type of sample |
| Modern* | INACR 57(b) | 0.62 | — | — | 0.67 | — | — | 33.9 | -3.0 | -5.7 |  |  |
| Modern* | INACR 50(a) | 0.60 | — | — | 0.65 | — | — | 38.5 | -3.1 | -6.4 |  |  |
| Modern* | INACR07 llA(a) | 0.62 | — | — | 0.67 | — | — | 33.9 | -0.5 | -6.3 |  |  |
| Modern* | INACR 50(b) | 0.60 | — | — | 0.65 | — | — | 38.5 | 2.0 | -3.8 |  |  |
| Modern* | INACR 8bBKl | 0.61 | — | — | 0.66 | — | — | 36.2 | -2.8 | -6.5 |  |  |
| Modern* | INARC 57(a) | 0.62 | — | — | 0.67 | — | — | 33.9 | -3.0 | -5.7 |  |  |
| Modern* | INACR 11(b) | 0.60 | — | — | 0.65 | — | — | 38.5 | -0.5 | -6.0 |  |  |
|  |  |  |  |  | 0.66 |  |  |  |  |  |  |  |
|  |  |  |  |  | 0.01 |  |  |  |  |  |  |  |
| ^L^70.6 – 65.5 | **347/LL** | 0.562 | 0.01 | 0.03 | 0.62 | -0.09 | 0.00 | 45.1 | -8.9 | -7.89 | 06 March 1992 | Gleb |
| ^L^70.6 – 65.5 | **L-1/3-4** | 0.603 | 0.02 | 0.03 | 0.66 | -0.83 | -0.08 | 35.1 | -10.8 | -7.51 | 05 February 2004 | Gleb |
| ^L^70.6 – 65.5 | **L-4/3-4** | 0.608 | 0.02 | 0.05 | 0.67 | -0.41 | -0.06 | 34.0 | -10.3 | -7.50 | 11 February 2004 | Gleb |
| ^L^70.6 – 65.5 | **L-2/3-4** | 0.634 | 0.02 | 0.04 | 0.70 | -0.38 | -0.08 | 28.3 | -9.3 | -7.46 | 05 February 2004 | Gleb |
| ^L^70.6 – 65.5 | **L-6/3-4** | 0.633 | 0.03 | 0.06 | 0.69 | -0.44 | -0.06 | 28.4 | -10.5 | -9.11 | 13 February 2004 | Gleb |
| ^L^70.6 – 65.5 | **351/LL** | 0.666 | 0.02 | 0.04 | 0.73 | -0.19 | 0.03 | 21.5 | -10.1 | -8.68 | 06 March 1992 | Gleb |
| ^L^70.6 – 65.5 | **341/LL** | 0.548 | 0.01 | 0.03 | 0.61 | -0.08 | 0.00 | 48.7 | -9.2 | -8.47 | 03 March 1992 | Gleb |
| ^L^70.6 – 65.5 | **353/LL** | 0.549 | 0.02 | 0.04 | 0.61 | -0.47 | -0.08 | 48.5 | -10.1 | -6.53 | 06 March 1992 | Gleb |
| ^L^70.6 – 65.5 | **L-9/3-4** | 0.582 | 0.02 | 0.03 | 0.64 | -0.30 | -0.12 | 40.0 | -10.2 | -8.44 | 16 February 2004 | Gleb |
| ^L^70.6 – 65.5 | **L-7/3-4** | 0.572 | 0.03 | 0.06 | 0.63 | -0.61 | -0.12 | 42.6 | -10.2 | -8.42 | 14 February 2004 | Gleb |
| ^L^70.6 – 65.5 | **L-8/3-4** | 0.554 | 0.03 | 0.06 | 0.62 | 0.32 | -0.09 | 47.0 | -9.2 | -12.96 | 15 February 2004 | Gleb |
| ^L^70.6 – 65.5 | **L-10/3-4** | 0.556 | 0.02 | 0.05 | 0.62 | -0.46 | -0.10 | 46.6 | -10.5 | -7.14 | 17 February 2004 | Gleb |
| ^L^70.6 – 65.5 | **L-5/3-4** | 0.563 | 0.01 | 0.03 | 0.62 | -0.45 | -0.10 | 44.8 | -10.3 | -7.05 | 12 February 2004 | Gleb |
| Average |  | 0.587 |  |  | 0.65 |  |  | 39.3 | -9.95 | -8.24 |  |  |
| Standard deviation |  | 0.039 |  |  | 0.04 |  |  | 9.0 | 0.61 | 1.60 |  |  |
| Standard error |  | 0.011 |  |  | 0.01 |  |  | 2.5 | 0.17 | 0.44 |  |  |
| ^B^145.5–125 | **^a^**BG-3/3-4 | 0.496 | 0.02 | 0.05 |  | -0.22 | -0.11 | — | — | — | 30 January 2004 | Rhizocretion |
| ^B^145.5–125 | **^a^**BG-2/9-0 | 0.696 | 0.02 | 0.04 |  | -0.64 | 0.00 | — | — | — | 04 February 2000 | Rhizo |
| ^B^145.5–125 | BG-9/9-0 | 0.608 | 0.02 | 0.05 | 0.67 | -0.24 | -0.03 | 34.1 | -7.16 | -6.06 | 09 February 2000 |  |
| ^B^145.5–125 | **^a^**BG-31/9-0 | 0.881 | 0.03 | 0.06 |  | -4.01 | 0.07 | — | — |  | 25 February 2000 |  |
| ^B^145.5–125 | **^a^**BG-6/9-0 | 0.512 | 0.02 | 0.05 |  | -0.92 | 0.02 | — | — |  | 09 February 2000 |  |
| ^B^145.5–125 | BG-3/9-0 | 0.586 | 0.02 | 0.04 | 0.65 | -0.63 | -0.04 | 39.2 | -6.73 | -5.66 | 04 February 2000 |  |
| ^B^145.5–125 | BG-21/9-0 | 0.569 | 0.01 | 0.02 | 0.63 | -0.66 | -0.02 | 43.3 | -4.81 | -3.02 | 16 February 2000 |  |
| ^B^145.5–125 | BG-10/9-0 | 0.538 | 0.02 | 0.04 | 0.60 | -0.77 | -0.07 | 51.4 | -6.86 | -5.71 | 09 February 2000 |  |
| ^B^145.5–125 | BG-8/9-0 | 0.626 | 0.02 | 0.03 | 0.69 | -0.31 | -0.04 | 29.9 | -7.04 | -6.32 | 09 February 2000 |  |
| ^B^145.5–125 | BG-5/9-0 | 0.562 | 0.01 | 0.03 | 0.62 | -0.56 | -0.08 | 45.0 | -5.80 | -5.92 | 09 February 2000 |  |
| ^B^145.5–125 | BG-32/9-0 | 0.606 | 0.03 | 0.07 | 0.67 | -0.86 | -0.03 | 34.4 | -5.61 | -2.05 | 25 February 2000 | Gleb |
| ^B^145.5–125 | BG-4/9-0 | 0.576 | 0.02 | 0.05 | 0.64 | -0.49 | -0.10 | 41.4 | -7.02 | -5.57 | 04 February 2000 |  |
| Average |  | 0.605 |  |  | 0.65 |  |  | 39.8 | -6.38 | -5.04 |  |  |
| Standard deviation |  | 0.102 |  |  | 0.03 |  |  | 7.0 | 0.86 | 1.58 |  |  |
| Standard error |  | 0.029 |  |  | 0.01 |  |  | 2.5 | 0.25 | 0.46 |  |  |
| ^D^245 –241 | D-6/3-4 | 0.692 | 0.02 | 0.04 | 0.75 | 0.38 | 0.14 | 16.4 | -10.49 | -5.94 | 25 January 2004 |  |
| ^D^245 –241 | D-24/3-4 | 0.678 | 0.02 | 0.06 | 0.74 | -1.18 | -0.07 | 19.1 | -5.34 | -3.95 | 28 January 2004 | Gleb |
| ^D^245 –241 | D-11/3-4 | 0.719 | 0.02 | 0.05 | 0.78 | -0.56 | -0.05 | 11.5 | -6.33 | -5.33 | 26 January 2004 |  |
| ^D^245 –241 | D-14/3-4 | 0.646 | 0.02 | 0.03 | 0.71 | 0.25 | 0.31 | 25.8 | -6.62 | -6.29 | 26 January 2004 | Crud |
| ^D^245 –241 | D-31/3-4 | 0.599 | 0.01 | 0.03 | 0.66 | 0.06 | -0.09 | 36.1 | -11.87 | -6.26 | 30 January 2004 |  |
| ^D^245 –241 | D-3/3-4 | 0.629 | 0.02 | 0.05 | 0.69 | -0.12 | -0.06 | 29.2 | -7.53 | -5.77 | 24 January 2004 |  |
| ^D^245 –241 | D-26/3-4 | 0.628 | 0.01 | 0.03 | 0.69 | -0.51 | -0.12 | 29.6 | -4.26 | -2.99 | 28 January 2004 | Plattey Gleb |
| ^D^245 –241 | D-13/3-4 | 0.664 | 0.03 | 0.06 | 0.73 | -1.00 | -0.10 | 22.0 | -8.72 | -5.04 | 26 January 2004 |  |
| ^D^245 –241 | D-7/3-4 | 0.610 | 0.02 | 0.03 | 0.67 | -0.61 | -0.07 | 33.4 | -11.25 | -6.15 | 26 January 2004 |  |
| ^D^245 –241 | D-6A/3-4 | 0.597 | 0.01 | 0.02 | 0.66 | -0.44 | -0.10 | 36.4 | -10.94 | -10.17 | 25 January 2004 |  |
| ^D^245 –241 | D-5/3-4 | 0.629 | 0.01 | 0.03 | 0.69 | 0.15 | -0.02 | 29.4 | -10.40 | -6.07 | 25 January 2004 | Rhizo |
| ^D^245 –241 | D-10/3-4 | 0.645 | 0.02 | 0.04 | 0.71 | -0.35 | -0.06 | 25.9 | -5.15 | -5.54 | 26 January 2004 |  |
| ^D^245 –241 | D-15/3-4 | 0.707 | 0.01 | 0.01 | 0.77 | -0.37 | -0.06 | 13.7 | -6.20 | -4.70 | 26 January 2004 |  |
| Average |  | 0.649 |  |  | 0.71 |  |  | 25.3 | -8.08 | -5.71 |  |  |
| Standard deviation |  | 0.040 |  |  | 0.04 |  |  | 8.2 | 2.65 | 1.66 |  |  |
| Standard error |  | 0.011 |  |  | 0.01 |  |  | 2.3 | 0.73 | 0.46 |  |  |
| ^P^250 –245 | P-14/2-3 | 0.648 | 0.02 | 0.04 | 0.71 | -1.03 | -0.05 | 25.2 | -6.68 | -1.90 | 23 January 2003 | Gleb/Rhizo |
| ^P^250 –245 | P-1/5-6 | 0.641 | 0.01 | 0.02 | 0.70 | -0.16 | 0.04 | 26.7 | -7.44 | -6.79 | 28 January 2006 | Gleb |
| ^P^250 –245 | P-13/2-3 | 0.727 | 0.01 | 0.03 | 0.79 | -0.54 | 0.15 | 10.0 | -11.22 | -3.97 | 23 January 2003 | Gleb |
| ^P^250 –245 | P-2/5-6 | 0.707 | 0.01 | 0.01 | 0.77 | -0.16 | 0.14 | 13.6 | -9.16 | -3.74 | 28 January 2006 | Gleb |
| ^P^250 –245 | **^a^**P-1/2-3 | 0.545 | 0.01 | 0.03 |  | -0.44 | -0.13 | — | — |  | 28 January 2003 | Gleb |
| ^P^250 –245 | P-3/2-3 | 0.644 | 0.02 | 0.05 | 0.71 | -0.99 | -0.10 | 26.2 | -7.41 | -3.07 | 18 January 2003 | Gleb |
| ^P^250 –245 | P-1/3-4 | 0.665 | 0.05 | 0.11 | 0.73 | 2.00 | 0.56 | 21.8 | -10.24 | -10.15 | 24 January 2004 | Gleb |
| ^P^250 –245 | **^a^**P-10/2-3 | 0.387 | 0.02 | 0.05 |  | 0.57 | -0.09 | — | — |  | 20 January 2003 | Rhizocretion |
| ^P^250 –245 | **^a^**P-2/3-4 | 0.485 | 0.02 | 0.05 |  | -0.26 | -0.13 | — | — |  | 24 January 2004 | Rhizo |
| ^P^250 –245 | **^a^**P-7/2-3 | 0.381 | 0.02 | 0.05 |  | -0.02 | -0.13 | — | — |  | 30 January 2003 |  |
| Average |  | 0.583 |  |  | 0.73 |  |  | 20.6 | -8.69 | -4.94 |  |  |
| Standard deviation |  | 0.127 |  |  | 0.04 |  |  | 7.1 | 1.80 | 3.02 |  |  |
| Standard error |  | 0.040 |  |  | 0.01 |  |  | 2.9 | 0.57 | 0.96 |  |  |
| ^M^271–266 | Mo-34A/5-6 | 0.606 | 0.02 | 0.03 | 0.67 | 1.36 | 0.05 | 34.4 | -7.83 | -15.63 | 23 January 2006 | Plates |
| ^M^271–266 | Mo-40A/5-6 | 0.620 | 0.01 | 0.01 | 0.68 | 0.14 | -0.08 | 31.3 | -7.91 | -11.48 | 24 January 2006 | Rhizocretion |
| ^M^271–266 | Mo-40B/5-6 | 0.602 | 0.02 | 0.04 | 0.66 | 0.03 | -0.10 | 35.3 | -6.61 | -11.36 | 24 January 2006 | Gleb/Rhizocretion |
| ^M^271–266 | Mo-34C/5-6 | 0.603 | 0.02 | 0.04 | 0.66 | 0.25 | -0.10 | 35.2 | -5.04 | -11.93 | 23 January 2006 | Rhizo |
| ^M^271–266 | **^a^**Mo-20C/5-6 | 0.417 | 0.01 | 0.03 |  | 0.12 | -0.10 | — | — |  | 21 January 2006 | Rhizo |
| ^M^271–266 | **^a^**Mo-34B/5-6 | 0.482 | 0.01 | 0.01 |  | -5.73 | -0.94 | — | — |  | 23 January 2006 | Nodule |
| Average |  | 0.555 |  |  | 0.67 |  |  | 34.0 | -6.85 | -12.60 |  |  |
| Standard deviation |  | 0.085 |  |  | 0.01 |  |  | 1.9 | 1.34 | 2.03 |  |  |
| Standard error |  | 0.035 |  |  | 0.00 |  |  | 0.9 | 0.55 | 0.83 |  |  |
| * Δ_47_ corresponded to heated gas | |  |  |  |  |  | | | | |  |  |
| ** Δ_47_ calibrated in CDES scale correction (Yoshida et l., 2013) | | | | | |  | | | | |  |  |
| ° Estimated δ^18^O for calcite from carbonate temperature precipitation (Kim and O'Neil, 1997) | | | | | | |  |  |  |  |  |  |
| **^a^**Anomaleous data: outliers from standard deviation, experimental contamination, diagenic effect | | | | | | |  |  |  |  |  |  |
| ^L,B,D,P,M^Bio-stratigraphic age of Lameta (Upper Cretaceous (Maastrichtian), Ghosh et al., 2003; Wilson et al., 2003; Shani and Khoshla 1994), Bagra (Early Cretaceous, modified after Ghosh et al., 2001; Bandyopadhaya and Sengupta, 2006; Bandyopadhaya, 2011), | | | | | | | | | | | | |
|
| Denwa (Early Mid. Triassic (Late Anisian), Bandyopadhaya & Sengupta, 2006; Bandyopadhaya, 2011), Pachmarhi (Early Triassic, Ghosh et al., 2012) and Motur (Early Mid Permian (Rodanian-Wordian), Ghosh et al., 2012; Ray and Chakraborty, 2002 ) formations | | | | | | | | | | | | |
|
